# Biological laterality and peripheral nerve DTI metrics

**DOI:** 10.1371/journal.pone.0260256

**Published:** 2021-12-16

**Authors:** Scott A. Holmes, Steven J. Staffa, Anastasia Karapanagou, Natalia Lopez, Victoria Karian, Ronald Borra, David Zurakowski, Alyssa Lebel, David Borsook

**Affiliations:** 1 Center for Pain and the Brain, Boston Children’s Hospital, Boston, Massachusetts, United States of America; 2 Department of Anesthesiology, Critical Care and Pain Medicine, Boston Children’s Hospital, Boston, Massachusetts, United States of America; 3 Department of Radiology, University Medical Center Groningen, University of Groningen, Groningen, Netherlands; 4 Department of Nuclear Medicine and Molecular Imaging, University Medical Center Groningen, University of Groningen, Groningen, Netherlands; INSERM, FRANCE

## Abstract

**Background and purpose:**

Clinical comparisons do not usually take laterality into account and thus may report erroneous or misleading data. The concept of laterality, well evaluated in brain and motor systems, may also apply at the level of peripheral nerves. Therefore, we sought to evaluate the extent to which we could observe an effect of laterality in MRI-collected white matter indices of the sciatic nerve and its two branches (tibial and fibular).

**Materials and methods:**

We enrolled 17 healthy persons and performed peripheral nerve diffusion weighted imaging (DWI) and magnetization transfer imaging (MTI) of the sciatic, tibial and fibular nerve. Participants were scanned bilaterally, and findings were divided into ipsilateral and contralateral nerve fibers relative to self-reporting of hand dominance. Generalized estimating equation modeling was used to evaluate nerve fiber differences between ipsilateral and contralateral legs while controlling for confounding variables. All findings controlled for age, sex and number of scans performed.

**Results:**

A main effect of laterality was found in radial, axial, and mean diffusivity for the tibial nerve. Axial diffusivity was found to be lateralized in the sciatic nerve. When evaluating mean MTR, a main effect of laterality was found for each nerve division. A main effect of sex was found in the tibial and fibular nerve fiber bundles.

**Conclusion:**

For the evaluation of nerve measures using DWI and MTI, in either healthy or disease states, consideration of underlying biological metrics of laterality in peripheral nerve fiber characteristics need to considered for data analysis. Integrating knowledge regarding biological laterality of peripheral nerve microstructure may be applied to improve how we diagnosis pain disorders, how we track patients’ recovery and how we forecast pain chronification.

## Introduction

Laterality is a biological state that may confer a preference for function of one side of the body vs. the other that occurs across mammalian species. In humans, lateralization of the left-brain hemisphere in functions such as speech, sensory and motor function, and language is observed in over 90% of individuals [1–3]. Lateralization of brain functions is linked to certain risk factors for addiction [4] and is a proposed means of optimizing brain performance [5] that may show a decline with age [6]. Such findings evince the behavioral and evolutionary advantages towards laterality of brain regions and neural networks in the central nervous system; however, it remains unclear if similar properties are observed in peripheral nerve fibers.

Peripheral nerve fiber conduction speed shows evidence of lateralization. Evaluations of peripheral nerve fibers is relevant for optimizing evaluation of clinical conditions such as small fiber neuropathies, myelinopathies, and other conditions that interrupt or attenuate the conduction of nerve signals. Beyond effects of age [7], prior investigations have evaluated the concept of laterality in peripheral nerve function and shown evidence to suggest bilateral differences in nerve fiber dynamics with potential domain specificity. For example, laterality differences in nerve fiber conduction speed along sensory nerve fibers have been observed [8, 9]; however, similar evaluations along motor neurons have observed no evidence of laterality [8, 10, 11]. More recently, the use of magnetic resonance imaging indices such as Diffusion Tensor Imaging (DTI) [12–16] as well as magnetization transfer ratio (MTR) imaging [17], have allowed for rapid evaluation of peripheral nerves in health and disease, producing reliable markers of nerve fiber integrity [16]. Findings restricted to FA and ADC have shown no evidence of lateralization in the sciatic nerve [18, 19]. However, these metrics are limited in their potential to comment on the microstructural properties of nerve tissue and may overlook variance attributable towards myelination, neurofilament density or edema, especially as nerve fibers further subdivide.

The sciatic nerve is the largest nerve in the human body and divides into the common peroneal/fibular and tibial branches. While an extensive literature exists on differences in muscle strength on lateralization [20–24], lateralization of the sciatic nerve and its branches has received a paucity of attention. Given that there are differences in muscles, it would be logical to expect differences in laterality of the sciatic nerve and its branches based on increased muscle use/load. In this paper we sought to define if (a) there was any evidence of laterality in any of the three nerve fiber bundles; and if so, (b) what DTI and MTR indices may be altered as measures of nerve fiber integrity. We hypothesized that there would be laterality differences in DTI and MTR metrics that would align with body side.

## Methods

### Human subjects

The study was approved by the ethics board at the Boston Children’s Hospital and subject experimentation was consistent with human pain studies noted in the Declaration of Helsinki. This study recruited participants from the Boston and surrounding areas. A total of 17 healthy controls were recruited. All individuals self-identified as right hand dominant with exception of one participant who reported being left-handed. Participants were grouped according to hand dominance. Inclusion criteria were healthy individuals aged 10–25. Exclusion criteria included: claustrophobia, significant medical problems (e.g., uncontrolled asthma, seizures, cardiac disorder), psychiatric problems, and other neurological disorders, pregnancy and any device or medical concern that would exclude subjects from having an MRI (e.g., magnetic implant, exceeding weight limit of scanner). Healthy controls were recruited from Boson and the surrounding areas through advertisements, and postings. All participants were compensated for their time. A detailed neurological examination was performed to ensure that subjects were otherwise healthy.

### Peripheral nerve imaging

#### Imaging acquisition

The scans were performed on a 3T Siemens Trio scanner located at Boston Children’s Hospital—Waltham. Imaging was performed using a 15-Channel knee coil. All subjects underwent conventional MRI scans (T1- and T2-weighted scans), reduced Field-of-View DTI, and MTI scans. The total imaging time was approximately 30 minutes for DTI and MTI acquisitions for each leg. DTI scanning parameters included: 20 diffusion directions, b = 750 s/mm^2^, voxel size = 0.8x0.8x5 mm^3^, axial slices = 28, TR/TE = 5200/103 ms, and 3 averages. Both scans were entered into the executed analyses. *MTI parameters included*: frequency offset = 1200 Hz, pulse duration = 9984μs, voxel size = 1.3x0.9x5 mm^3^, axial slices = 28, TR/TE = 1190/4.37ms, flip angle = 20°, bandwidth = 380Hz/Px, and 2 averages.

#### Image processing

**Fig 1:**
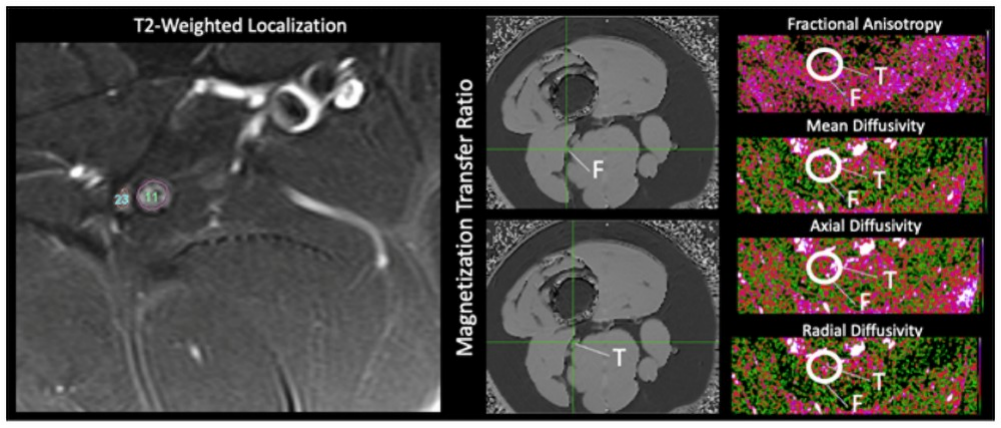
Regions of interest. Overview of how regions of interest were identified in the tibial and fibular nerve divisions using T2 Weighted Localization, and how the Tibial and Fibular nerves appeared on MTR and DWI sequences.

Data analysis was performed using Olea SphereTM V2.3 (Olea Medical^®^). Regions of interest were drawn manually at the sciatic, tibial and fibular nerves on the T2-weighted images and were supervised by RB, (see Fig 1). Regions of interest were extracted to evaluate the degree of isotropic motion of water within nerve segments, and therein characterize the integrity of white matter architecture [25]. Fractional anisotropy (FA), mean diffusivity (MD), radial diffusivity (RD), axial diffusivity (AD), were calculated using the Olea Sphere software. Magnetization transfer ratio (MTR) values were calculated from the ratio of on- and off-resonance sequences and reported for each nerve and reflect relative differences in factors such as myelination [26]. Multiple slices were collected for each nerve division in each participant. Both mean data as well as variance data were collected from each nerve division reflecting within slice averages and standard deviations of nerve fiber characteristics.

### General statistical considerations

DTI parameters are presented using means and standard deviations stratified by nerve type (sciatic, tibial and fibular). Leg data was divided based on reported hand-dominance. For example, if a participant reported to be right-handed, the left leg was contralateral and right leg was ipsilateral. Multivariable linear regression analysis using Generalized Estimating Equations (GEE) modeling with repeated measures was applied to determine the independent association between leg laterality (ipsilateral vs. contralateral) and each continuous DTI parameter accounting for multiple scans within each subject adjusting for age and sex as covariates. An identity link function and Gaussian distribution was used in statistical analysis with compound symmetry correlation to handle the multiple MRI scans from the same subject. Results from GEE modeling are presented as adjusted differences between contralateral and ipsilateral legs with corresponding 95% confidence intervals and p-values. A Bonferroni-adjusted statistical significance threshold of p < 0.003 (0.05/15) was implemented to minimize the risk of false-positive results (type I error) due to multiple testing across the 3 nerve types. Statistical analyses were performed using Stata software (version 15.0, StataCorp LLC, College Station, Texas).

## Results

**Fig 2:**
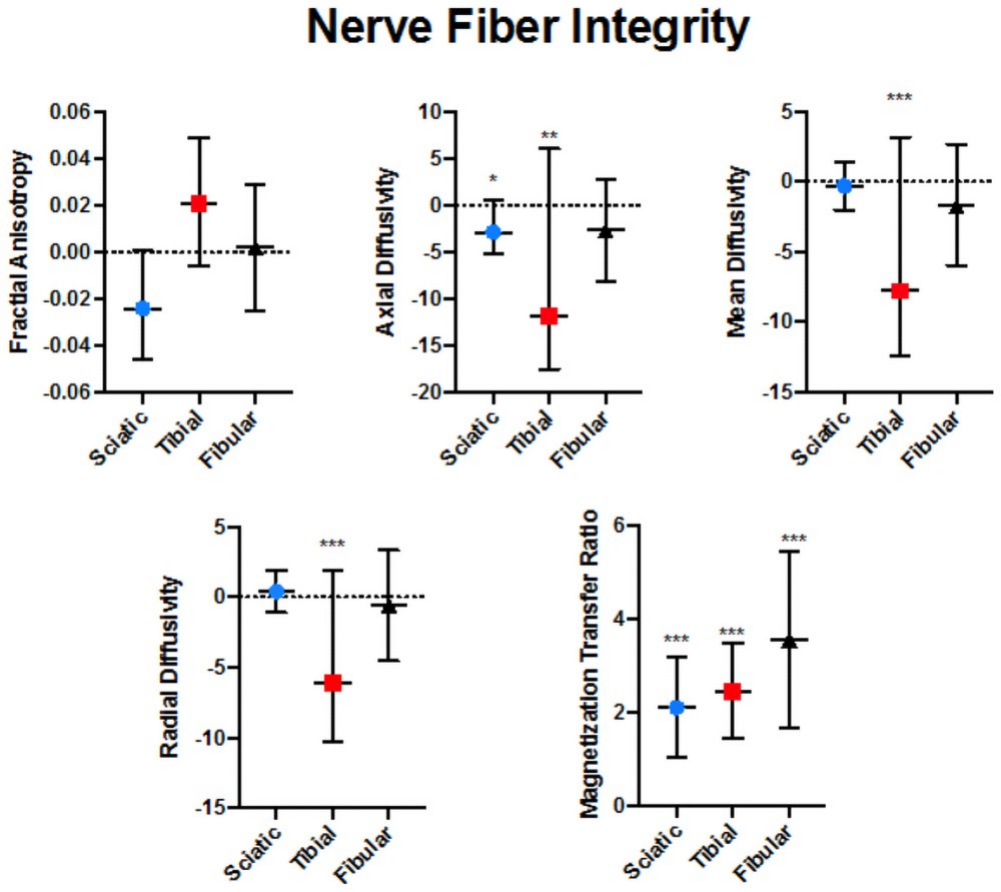
Nerve fiber integrity. Adjusted differences reflecting ipsilateral measures minus contralateral measures are plotted with confidence intervals values in the three evaluated nerves. * represents a corrected p-value < 0.003; ***p<0.001 (Bonferroni-adjusted for multiple comparisons).

Participants were on average 17.24 years of age (SD = 4.24) and included eight females and nine males. A total of 1051 slices were used for analysis divided between sciatic (n = 493), tibial, (n = 277) and fibular (n = 281) nerve divisions. Mean values for all DTI and MTI comparisons are provided in Table 1. Significant differences were observed for laterality in all three nerve fiber divisions in terms of mean and variance metrics (see Table 2 and Fig 2). In the sciatic nerve, significantly higher mean AD was found in the contralateral nerve bundle whereas higher mean MTR was found in the ipsilateral nerve bundle. Mean FA and the variance of MD both approached significance (p<0.05) but were not statistically significant. In the tibial nerve, higher levels of RD, AD, and MD were found in the contralateral nerve bundle leg and greater MTR levels were consistently observed in the ipsilateral nerve bundle leg. These findings were observed for both mean and variance values. For the Fibular nerve, mean MTR was greater in the ipsilateral than the contralateral nerve bundle.

**Table 1:** Raw means and standard deviations from MTI and DWI metrics in each nerve bundle for the contralateral and ipsilateral legs. Mean data as well as variance data were collected from each nerve division reflecting within slice averages and standard deviations of nerve fiber characteristics.

	Sciatic Nerve		Tibial Nerve		Fibular Nerve	
Variable	Contralateral	Ipsilateral	Contralateral	Ipsilateral	Contralateral	Ipsilateral
FA Mean	0.54 (0.14)	0.56 (0.14)	0.42 (0.15)	0.42 (0.14)	0.40 (0.15)	0.40 (0.15)
FA Variance	0.25 (0.03)	0.24 (0.05)	0.23 (0.04)	0.23 (0.04)	0.23 (0.05)	0.22 (0.04)
RD Mean	24.86 (9.87)	25.41 (10.08)	35.43 (18.22)	41.96 (18.11)	35.48 (17.23)	36.73 (18.27)
RD Variance	19.07 (4.67)	20.12 (4.59)	20.90 (5.55)	23.90 (5.21)	22.42 (6.90)	20.36 (5.69)
AD Mean	52.05 (18.25)	57.28 (17.68)	60.76 (25.46)	73.04 (23.22)	56.39 (23.72)	60.03 (24.94)
AD Variance	25.64 (7.11)	27.96 (6.92)	27.93 (7.92)	32.84 (7.35)	28.14 (9.46)	27.04 (8.74)
MD Mean	33.54 (12.45)	35.29 (12.50)	43.98 (20.45)	52.15 (19.41)	41.80 (18.84)	44.28 (20.43)
MD Variance	21.20 (5.33)	22.24 (5.39)	23.00 (6.02)	26.40 (5.61)	24.35 (7.72)	22.40 (6.58)
MTR Mean	29.19 (6.11)	35.33 (5.70)	38.12 (3.72)	32.60 (3.96)	32.88 (8.39)	36.51 (5.89)
MTR Variance	11.15 (5.36)	10.14 (3.44)	12.64 (3.57)	10.74 (3.48)	11.48 (2.98)	11.55 (2.95)

**Table 2:** Comparison of ipsilateral vs. contralateral legs. Values reflect the mean and variance of within slice parameters.

Comparison of Dominant Leg vs Non-dominant Legs									
	Sciatic Nerve			Tibial Nerve			Fibular Nerve		
Variable	Adjusted difference (Ipsilateral—Contralateral)	95% CI	P value	Adjusted difference (Ipsilateral—Contralateral)	95% CI	P value	Adjusted difference (Ipsilateral—Contralateral)	95% CI	P value
**FA mean**	-0.024	(-0.046, -0.001)	0.042	0.021	(-0.006, 0.049)	0.132	0.002	(-0.025, 0.029)	0.872
**FA variance**	-0.009	(-0.018, 0.004)	0.199	0.008	(-0.004, 0.019)	0.181	0.007	(-0.007, 0.021)	0.324
**RD mean**	0.41	(-1.08, 1.91)	0.587	-6.12	(-10.31, -1.93)	**0.004** *	-0.58	(-4.52, 3.35)	0.769
**RD variance**	-0.69	(-1.69, 0.31)	0.178	-3.32	(-4.33, -2.31)	**<0.001** *	1.43	(-0.28, 3.13)	0.101
**AD mean**	-2.86	(-5.13, -0.59)	**0.014** *	-11.77	(-17.48, -6.07)	**<0.001** *	-2.65	(-8.09, 2.78)	0.338
**AD variance**	-2.84	(-4.09, -1.60)	**<0.001** *	-6.13	(-7.63, -4.64)	**<0.001** *	0.25	(-1.97, 2.48)	0.822
**MD mean**	-0.3	(-2.02, 1.41)	0.729	-7.77	(-12.39, -3.15)	**0.001** *	-1.67	(-5.98, 2.66)	0.448
**MD variance**	-1.21	(-2.23, -0.18)	0.021	-3.99	(-5.04, -2.94)	**<0.001** *	1.09	(-0.72, 2.9)	0.236
**MTR mean**	2.11	(1.04, 3.19)	**<0.001** *	2.46	(1.45, 3.48)	**<0.001** *	3.56	(1.68, 5.45)	**<0.001** *
**MTR variance**	0.29	(-0.47, 1.06)	0.451	2.77	(1.66, 3.87)	**<0.001** *	0.26	(-0.79, 1.32)	0.627

GEE modeling was used to account for multiple measurements per patient, while adjusting for age, sex and scan number.

CI; confidence interval.

* statically significant p <0.017 (Bonferroni-adjusted).

An exploratory analysis of sex and age was performed. Overall, variance in FA of the sciatic nerve was the only nerve imaging factor associated with age (adjusted coefficient per year of age: 0.002; CI: 0.0005, 0.004; p-value: 0.014). For sex, males had higher values of RD, MD, and AD than females; whereas females had larger values FA in terms of mean and variance than males.

## Discussion

Biological laterality is intimately tied to functional performance. In particular, laterality may infer a preference that is present in humans for use of one side of the body vs. the other that confers an evolutionary and behavioral advantage [6]. While some functions are heavily or uniquely lateralized such as speech and language, peripheral lateralization of nerve fibers is not a frequent measure in studies of sensory or motor evaluation. Here we evaluated the microstructural integrity of lower extremity nerves in both legs in a cohort of healthy subjects as part of a larger study following nerve injury due to ankle sprain [25]. The field of view was focused on the sciatic nerve before and after its division into the tibial and fibular nerve. Differences based on laterality in DTI metrics were observed in the sciatic and tibial nerves whereas MTR differences were found in each nerve division.

### Differences in laterality of nerve fiber characteristics

Laterality shows an influence on the microstructural integrity of peripheral nerves. Prior research evaluating peripheral nerve fibers has alluded to possible lateralization based on nerve function and found inconsistent results. These studies have shown that in functionally dominant nerve fibers, an increase in conduction velocity is observed and is focused on sensory nerve conduction velocities [26], giving reason to suspect potential changes in nerve structure as well. Investigations into the sciatic nerve have reported no differences based on laterality [18, 19] using FA and ADC. Their findings would align with ours in that we also did not observe a laterality difference in the majority of DTI parameters within the sciatic nerve bundle. However, our work extends their findings suggesting that nerve fiber characteristics appear to become more unique in more distal divisions of peripheral nerve fibers. Moreover, greater evidence of a sex effect was observed in the tibial and fibular nerve divisions, further underscoring the proximal-distal effect. In the current investigation, we show evidence of increased AD, RD, and MD in the tibial nerve; which points towards an increase in the isotropic nature of water diffusion in contralateral versus ipsilateral nerve fiber bundles [27]. We also observe similar trends in within slice variability, particularly within the tibial nerve division. The reasons for observing a laterality difference–in a healthy cohort–may pertain to a relatively lower presence of microstructural tissue, both within and external to the nerve fiber, which may be accounted for by factors such as myelination [28], nerve fiber density [29], and level of edema [30]. This suggests that peripheral nerve DTI may be sensitive to demonstrating increased myelination of nerve fibers ipsilateral to hand dominance and would be supported by findings from MTR, where lower MTR values in the contralateral sciatic, tibial and fibial nerve divisions were observed. Notably, the use of MTR has largely been applied towards showing changes in level of myelination in myelopathies and is sensitive to attempts of both de- and remyelination [31]. The consistent findings of MTR across each nerve fiber bundle would agree with this interpretation. Together, current findings provide a clear demonstration that there is a unique DTI and MTR profile based on laterality of nerve fibers that may specifically relate to their level of myelination and level of division.

### Functionally mediated differentiation

Differences in characteristics of bodily tissue are functionally mediated. This appears to be true as well for peripheral nerve fibers. Prior research has proposed that lateralization of the human brain is functionally based and observed as a means of optimizing behavioral performance [5]. This beneficent organization is attenuated with age as neuroplastic mechanisms become less efficient [6]. It would be logical that this organization of nerve structures following function is not simply a concept applied towards central rather than peripheral organization but adopted globally. Indeed, current results would align with this proposal when considering that more distal structures offer more opportunity for lateralization than proximal structures. The sciatic nerve, emerging from the sacral plexus of the lumbar and sacral regions of the spinal column, innervates muscle structures in the thigh and hips [32]. Alternatively, tibial and fibular divisions innervate relatively fine motor structures. While the tibial nerve innervates muscles of the lower leg associated with plantar flexion and locomotion [33], the fibular nerve innervates muscles implicated in balance and ankle stability [34]. As such, with more opportunity for functional differentiation, there may be greater potential for lateralization. Observing effects predominately in the tibial, rather than fibular nerves may align with the former innervating muscles used during gait power (e.g., gastrocnemius and soleus) aligning with functional asymmetries observed in prior work and the latter supporting balance, requiring more symmetry between legs. Findings from the MTR analyses supporting greater myelin in nerve fibers ipsilateral to the dominant hand align with this functional interpretation as greater myelin levels is associated with greater nerve fiber conductions speed [35, 36]. As such, findings suggest differences in peripheral nerve fiber laterality may relate to particular nerve divisions and be functionally mediated as in the brain.

Integrating current findings into future research will be of critical importance as peripheral nerve imaging is a relatively new and evolving field. In particular, current findings argue that supporting neuronal architecture is unique to each leg, which merits corresponding investigations using techniques such as dual-energy x-ray absorptiometry (DEXA) to understand if nerve fiber characteristics relate to, or mediate muscle and bone structure. Clinically, current findings support the need to control for which leg is evaluated when attempting to address peripheral neuropathies from chemotherapy [37], diabetic peripheral neuropathy [38], or injury [25] on the peripheral nervous system, and urge caution when using contralateral nerve fibers as internal controls. Moreover, our exploration of study findings underscores a potential impact of sex, furthering the notion that findings relate to nerve function to support a ‘generally’ greater muscle mass, as is generally seen when comparing males relative to females [39].

### Analytical issues

Technology surrounding peripheral nerve imaging is currently limited. Although preliminary work has identified factors such as demographics and body type [15] that may mediate peripheral nerve fiber characteristics, analytical methods are restrained from basic factors such as observer bias as we depend on manual segmentation of nerve fibers. Current techniques for evaluation of nerve fiber health with diffusion weighted metrics is limited to larger nerve bundles based on resolution; however, small nerve fiber distributions can be objectively evaluated using coarser techniques such as microscopy [40, 41] that provide external metrics such as fiber counts and degree of arborization. Ideally, the field will proceed towards automation which will require agreement on acquisition parameters, and analysis tools. Factors that limit peripheral nerve imaging are focused on imaging off isocenter in the body and therefore address magnetic field inhomogeneity, motion, fat suppression, aliasing and distortion [13]. Development of higher field MRIs, more sensitive acquisition parameters and radio-frequency coils for analysis will help transition our ability to evaluate smaller nerve fibers internally with the same objectivity we apply towards larger nerve fiber groups.

### Caveats

There are a few caveats that should be mentioned. In this cohort, we separated groups based on their self-reported hand dominance. As such, we are limited in our ability to infer the functional dominance of each leg. These will be the subject of future research as we expand our program on peripheral neurography. Weight and height have been correlated with DTI metrics in addition to age in pediatric subjects [42]. Although we did control for age, we did not record either weight or height and the independent correlation of weight, height and age with DTI remains unclear. We proposed a functionally mediated interpretation of peripheral nerve laterality extending from research into the central nervous system and muscle fiber distribution. Future research should formally evaluate the congruence of these observations, as well as evaluate the impact of factors such as age to determine the proximity of our work to that of Esteves and colleagues [6]. It would be of value both for basic science and clinical research to begin developing normative data [14, 43] for DTI and MTR parameters in peripheral nerve tissue.

### Conclusions

Lateralization is a concept promoted by the human body to optimize behavioral performance and is observed across bodily systems. This concept has been demonstrated at the level of brain circuitry, muscle development, and sensory processing. Findings from this investigation replicate and extend prior work on peripheral nerve fiber evaluation by showing how differences in the microstructural properties of peripheral nerves can be quantified in a reliable way and how they vary according to nerve division. Therefore, it is important to integrate laterality into investigations of peripheral nerve structure.

## References

[1] Costanzo EY, Villarreal M, Drucaroff LJ, Ortiz-Villafañe M, Castro MN, Goldschmidt M, et al. Hemispheric specialization in affective responses, cerebral dominance for language, and handedness. Behav Brain Res. 2015;288: 11–19. doi: 10.1016/j.bbr.2015.04.006 25882724

[2] Hodgson JC, Hudson JM. Speech lateralization and motor control. Progress in Brain Research. Elsevier; 2018. pp. 145–178. doi: 10.1016/bs.pbr.2018.06.009 30097191

[3] Sainburg RL. Convergent models of handedness and brain lateralization. Front Psychol. 2014;5. doi: 10.3389/fpsyg.2014.01092 25339923 PMC4189332

[4] Gordon HW. Laterality of Brain Activation for Risk Factors of Addiction. Curr Drug Abuse Rev. 2016;9: 1–18. doi: 10.2174/1874473709666151217121309 26674074 PMC4811731

[5] Esteves M, Moreira PS, Marques P, Castanho TC, Magalhães R, Amorim L, et al. Asymmetrical subcortical plasticity entails cognitive progression in older individuals. Aging Cell. 2019;18. doi: 10.1111/acel.12857 30578611 PMC6351824

[6] Esteves M, Ganz E, Sousa N, Leite-Almeida H. Asymmetrical Brain Plasticity: Physiology and Pathology. Neuroscience. 2020; S0306452220300427. doi: 10.1016/j.neuroscience.2020.01.022 32027996

[7] Mallik A. Nerve conduction studies: essentials and pitfalls in practice. J Neurol Neurosurg Psychiatry. 2005;76: ii23–ii31. doi: 10.1136/jnnp.2005.069138 15961865 PMC1765692

[8] Gupta N, Sanyal S, Babbar R. Sensory nerve conduction velocity is greater in left handed persons. Indian J Physiol Pharmacol. 2008;52: 189–92. 19130864

[9] Patel A, Mehta A. A Comparative Study Of Nerve Conduction Velocity Between Left And Right Handed Subjects. 2014; 5. PMC4206210 25346929

[10] Bhorania S, Ichaporia R. Effect of limb dominance on motor nerve conduction. INDIAN J PHYSIOL PHARMACOL. 2009;53: 279–82. 20329377

[11] Tsuji Y, Noto Y, Shiga K, Yokota I, Nakagawa M, Mizuno T. Does hand dominance affect peripheral nerve excitability? Clin Neurophysiol. 2016;127: 1921–1922. doi: 10.1016/j.clinph.2016.01.009 26971471

[12] Heckel A, Weiler M, Xia A, Ruetters M, Pham M, Bendszus M, et al. Peripheral Nerve Diffusion Tensor Imaging: Assessment of Axon and Myelin Sheath Integrity. Yang J, editor. PLOS ONE. 2015;10: e0130833. doi: 10.1371/journal.pone.0130833 26114630 PMC4482724

[13] Jeon T, Fung MM, Koch KM, Tan ET, Sneag DB. Peripheral nerve diffusion tensor imaging: Overview, pitfalls, and future directions: Challenges of DTI in the Periphery. J Magn Reson Imaging. 2018;47: 1171–1189. doi: 10.1002/jmri.25876 29083521

[14] Kakuda T, Fukuda H, Tanitame K, Takasu M, Date S, Ochi K, et al. Diffusion tensor imaging of peripheral nerve in patients with chronic inflammatory demyelinating polyradiculoneuropathy: a feasibility study. Neuroradiology. 2011;53: 955–960. doi: 10.1007/s00234-010-0833-z 21318578

[15] Kronlage M, Schwehr V, Schwarz D, Godel T, Uhlmann L, Heiland S, et al. Peripheral nerve diffusion tensor imaging (DTI): normal values and demographic determinants in a cohort of 60 healthy individuals. Eur Radiol. 2018;28: 1801–1808. doi: 10.1007/s00330-017-5134-z 29230526

[16] Simon NG, Lagopoulos J, Gallagher T, Kliot M, Kiernan MC. Peripheral nerve diffusion tensor imaging is reliable and reproducible: Reliability of Peripheral Nerve DTI. J Magn Reson Imaging. 2016;43: 962–969. doi: 10.1002/jmri.25056 26397723

[17] Kollmer J, Kästel T, Jende JME, Bendszus M, Heiland S. Magnetization Transfer Ratio in Peripheral Nerve Tissue: Does It Depend on Age or Location? Invest Radiol. 2018;53: 397–402. doi: 10.1097/RLI.0000000000000455 29470194

[18] Wada K, Hashimoto T, Miyagi R, Sakai T, Sairyo K. Diffusion tensor imaging and tractography of the sciatic nerve: assessment of fractional anisotropy and apparent diffusion coefficient values relative to the piriformis muscle, a preliminary study. Skeletal Radiol. 2017;46: 309–314. doi: 10.1007/s00256-016-2557-6 28028573

[19] Wako Y, Nakamura J, Eguchi Y, Hagiwara S, Miura M, Kawarai Y, et al. Diffusion tensor imaging and tractography of the sciatic and femoral nerves in healthy volunteers at 3T. J Orthop Surg. 2017;12: 184. doi: 10.1186/s13018-017-0690-0 29187253 PMC5707804

[20] Demura S, Sato S, Minami M. Utility of an ADL index for institutionalized elderly people: Examining possible applications for independent elderly people. 2001; 8. 10.1007/BF02897307 PMC2723652 21432235

[21] Demura S, Miyaguchi K, Aoki H. The difference in output properties between dominant and nondominant limbs as measured by various muscle function tests. J Strength Cond Res. 2010;24: 2816–20. doi: 10.1519/JSC.0b013e3181e38293 20885200

[22] Noguchi T, Demura S, Takahashi K, Demura G, Mori Y. Differences in Muscle Power Between the Dominant and Nondominant Upper Limbs of Baseball Players. J Strength Cond Res. 2014;28: 82–86. doi: 10.1519/JSC.0b013e3182909112 23524366

[23] Oppewal A, Hilgenkamp TIM, van Wijck R, Evenhuis HM. The effect of handedness on grip strength in older adults with intellectual disabilities. Res Dev Disabil. 2013;34: 1623–1629. doi: 10.1016/j.ridd.2013.02.013 23475012

[24] Provins K, Magliaro J. Skill, strength, handedness, and fatigue. J Mot Behav. 1989;21: 113–21. doi: 10.1080/00222895.1989.10735469 15132940

[25] Holmes S, Barakat N, Bhasin M, Lopez NI, Lebel A, Zurakowski D, et al. Biological and behavioral markers of pain following nerve injury in humans. Neurobiol Pain. 2020;7: 100038. doi: 10.1016/j.ynpai.2019.100038 31890990 PMC6926375

[26] Tan U. Sensory nerve conduction veleocities are higher on teh left than the right hand and motor conduction is faster on teh right hand than left in right-handed normal subjects. Int J Neurosci. 1993;73: 85–91. doi: 10.3109/00207459308987214 8132422

[27] Winklewski PJ. Understanding the Physiopathology Behind Axial and Radial Diffusivity Changes—What Do We Know? Front Neurol. 2018;9: 6. doi: 10.3389/fneur.2018.00006 29535676 PMC5835085

[28] Song S-K, Sun S-W, Ju W-K, Lin S-J, Cross AH, Neufeld AH. Diffusion tensor imaging detects and differentiates axon and myelin degeneration in mouse optic nerve after retinal ischemia. NeuroImage. 2003;20: 1714–1722. doi: 10.1016/j.neuroimage.2003.07.005 14642481

[29] Wright DK, Johnston LA, Kershaw J, Ordidge R, O’Brien TJ, Shultz SR. Changes in Apparent Fiber Density and Track-Weighted Imaging Metrics in White Matter following Experimental Traumatic Brain Injury. J Neurotrauma. 2017;34: 2109–2118. doi: 10.1089/neu.2016.4730 28152648

[30] Chen Y-Y, Zhang X, Lin X-F, Zhang F, Duan X-H, Zheng C-S, et al. DTI metrics can be used as biomarkers to determine the therapeutic effect of stem cells in acute peripheral nerve injury: DTI of Stem Cell Therapy in Nerve Injury. J Magn Reson Imaging. 2017;45: 855–862. doi: 10.1002/jmri.25395 27448779

[31] Giacomini PS, Levesque IR, Ribeiro L, Narayanan S, Francis SJ, Pike GB, et al. Measuring Demyelination and Remyelination in Acute Multiple Sclerosis Lesion Voxels. Arch Neurol. 2009;66. doi: 10.1001/archneurol.2008.578 19273757

[32] Giuffre B, Jeanmonod R. Anatomy, Sciatic Nerve. Stats Pearls; 2021. 29494038

[33] Desai S, Cohen-Levy W. Anatomy, Bony Pelvis and Lower Limb, Tibial Nerve. Stats Pearls; 2020. 30725713

[34] Van den Bergh FRA, Vanhoenacker FM, De Smet E, Huysse W, Verstraete KL. Peroneal nerve: Normal anatomy and pathologic findings on routine MRI of the knee. Insights Imaging. 2013;4: 287–299. doi: 10.1007/s13244-013-0255-7 23709403 PMC3675257

[35] Sato A, Sato Y, Suzuki H. Aging effects on conduction velocities of myelinated and unmyelinated fibers of peripheral nerves. 1985; 6. 10.1016/0304-3940(85)90090-4 3991047

[36] Waxman SG. Conduction in Myelinated, Unmyelinated, and Demyelinated Fibers. Arch Neurol. 1977;34: 585–589. doi: 10.1001/archneur.1977.00500220019003 907529

[37] Kerckhove N, Collin A, Condé S, Chaleteix C, Pezet D, Balayssac D. Long-Term Effects, Pathophysiological Mechanisms, and Risk Factors of Chemotherapy-Induced Peripheral Neuropathies: A Comprehensive Literature Review. Front Pharmacol. 2017;8. doi: 10.3389/fphar.2017.00086 28286483 PMC5323411

[38] Selvarajah D, Kar D, Khunti K, Davies MJ, Scott AR, Walker J, et al. Diabetic peripheral neuropathy: advances in diagnosis and strategies for screening and early intervention. Lancet Diabetes Endocrinol. 2019;7: 938–948. doi: 10.1016/S2213-8587(19)30081-6 31624024

[39] Schorr M, Dichtel LE, Gerweck AV, Valera RD, Torriani M, Miller KK, et al. Sex differences in body composition and association with cardiometabolic risk. Biol Sex Differ. 2018;9: 28. doi: 10.1186/s13293-018-0189-3 29950175 PMC6022328

[40] Leckenby JI, Chacon MA, Grobbelaar AO, Lichtman JW. Imaging Peripheral Nerve Regeneration: A New Technique for 3D Visualization of Axonal Behavior. J Surg Res. 2019;242: 207–213. doi: 10.1016/j.jss.2019.04.046 31085369

[41] Utsunomiya T, Nagaoka T, Hanada K, Omae T, Yokota H, Abiko A. Imaging of the corneal subbasalwhorl-likenerve plexus: More accurate depiction of the extent of corneal nerve dmage in patients with diabetes. Invest Ophthalmol Vis Sci. 2015;56: 5417–23. doi: 10.1167/iovs.15-16609 26284545

[42] Hofstadler B, Bäumer P, Schwarz D, Kronlage M, Heiland S, Bendszus M, et al. MR Neurography: Normative Values in Correlation to Demographic Determinants in Children and Adolescents. Clin Neuroradiol. 2020;30: 671–677. doi: 10.1007/s00062-019-00834-9 31486885

[43] Hilly O, Chen JM, Birch J, Hwang E, Lin VYW, Aviv RI, et al. Diffusion Tensor Imaging Tractography of the Facial Nerve in Patients With Cerebellopontine Angle Tumors. Otol Neurotol. 2016;37: 388–393. doi: 10.1097/MAO.0000000000000984 26905823

